# Functional interpretation of non-coding sequence variation: Concepts and challenges

**DOI:** 10.1002/bies.201300126

**Published:** 2013-12-05

**Authors:** Dirk S Paul, Nicole Soranzo, Stephan Beck

**Affiliations:** 1) UCL Cancer Institute, University College LondonLondon, United Kingdom; 2) Wellcome Trust Sanger InstituteHinxton, Cambridge, United Kingdom; 3) Department of Haematology, University of CambridgeCambridge, United Kingdom

**Keywords:** complex traits, chromatin, genome editing, gene regulation, GWAS, regulatory variants

## Abstract

Understanding the functional mechanisms underlying genetic signals associated with complex traits and common diseases, such as cancer, diabetes and Alzheimer's disease, is a formidable challenge. Many genetic signals discovered through genome-wide association studies map to non-protein coding sequences, where their molecular consequences are difficult to evaluate. This article summarizes concepts for the systematic interpretation of non-coding genetic signals using genome annotation data sets in different cellular systems. We outline strategies for the global analysis of multiple association intervals and the in-depth molecular investigation of individual intervals. We highlight experimental techniques to validate candidate (potential causal) regulatory variants, with a focus on novel genome-editing techniques including CRISPR/Cas9. These approaches are also applicable to low-frequency and rare variants, which have become increasingly important in genomic studies of complex traits and diseases. There is a pressing need to translate genetic signals into biological mechanisms, leading to prognostic, diagnostic and therapeutic advances.

## Introduction

The quest for identifying sequence variants associated with complex traits including common diseases has been greatly facilitated by technological progress in high-throughput DNA analysis, such as genotyping arrays and next-generation sequencing, complemented by advances in bioinformatics 1. In recent years, researchers have systematically assayed millions of common genetic variants across hundreds of thousands of individuals in genome-wide association studies (GWAS). In GWAS, the allele frequencies of a set of sequence variants are statistically compared between individuals with a phenotype of interest (such as a clinical condition) and the general population. This results in the detection of sequence variants that show association with the phenotype. Despite the remarkable success of GWAS, there is a substantial gap between the plethora of associated sequence variants and our understanding of how most of these variants contribute to complex trait biology 2–4. At least three key issues have impeded the functional translation of GWAS signals.

First, GWAS have focused on common SNPs (MAF > 5%). SNPs either individually or in combination typically explain only a small fraction of the genetic variance of most complex traits 5,6. As a consequence, phenotypic effects due to the perturbation of trait-associated SNPs are likely to be subtle. This implies that large numbers of independent studies are required to estimate quantitatively the phenotypic impact of each genetic variant. Furthermore, highly sensitive assays in sufficiently large sample sizes may be required for their downstream assessment and validation.

Second, the associated sequence variant identified in GWAS may in fact only be linked to, rather than itself be, the causal variant. This phenomenon is known as linkage disequilibrium (LD) 7. The alleles of the index (lead) SNP, i.e. the sequence variant showing the strongest association with a trait of interest, are correlated with the alleles of multiple nearby proxy SNPs. The combination of these alleles form haplotypes along the chromosome and are transmitted together. Importantly, such haplotype structures are population-specific 8–10. On genotyping platforms, only a few selected index SNPs per LD region are measured. In fact, the platforms exploit LD patterns in a way that the selected SNPs capture most of the genetic variation at any given locus 8. In individuals of Northern European ancestry, LD structure extends to around 50 kilobases (with substantial variation 10) and usually harbors several genes and transcripts. Because of LD, it may not be possible to discriminate statistically between multiple variants associated with a phenotype at a determined locus. As discussed in this review, the identification of suitable functional genome maps may be particularly helpful in prioritizing efforts for these loci.

Third, the vast majority (over 90%) of associated variants have been found to localize outside of known protein-coding sequences, thus impeding the direct interpretation of their functional effects 11. To this end, the trait-associated variant may be involved in regulation of gene expression, which is chiefly dependent on cell type identity, developmental stage and environmental factors 12. The variant may reside at gene regulatory elements, such as promoters, enhancers, silencers, and insulators, where it perturbs binding sites of transcription factors, local chromatin structure or co-factor recruitment, ultimately resulting in changes of transcriptional output of the nearby gene(s) 12. Trait-associated variants that are located distal to transcription start sites at gene-dense regions are particularly difficult to interpret. Here, literature search or more precisely, experimental validation may determine selection of a candidate gene. In our article, we mainly discuss the influence of sequence variation on regulatory elements of protein-coding genes, but we recognize that sites affecting the transcription of non-protein coding RNAs may also play an important role in gene regulation.

The challenge ahead is to carve out suitable strategies to gain insights into cell type-specific molecular processes and pathways underlying the discovered GWAS signals. In this article, we (i) describe principles for interpreting non-coding genetic signals using public genome annotation resources; (ii) discuss considerations when using annotation data sets in primary cells, cell lines, and – looking ahead – differentiated induced pluripotent stem cells (iPSCs); and (iii) outline current and emerging strategies to prioritize and experimentally validate candidate regulatory variants and genes.

## Genome annotation resources can guide the interpretation of genetic variation

The genomic positions of GWAS index SNPs and proxy SNPs can be compared to the positions of biochemical events referenced in publicly available annotation resources to help tease out the functional variant(s) from the vast number of trait-associated variants in LD. Such biochemical events include sites of transcription factor and micro RNA (miRNA) binding, chromatin accessibility and modifications, DNA methylation, and many other types. A trait-associated variant that overlaps with a regulatory element may be functionally relevant. The overlap also directly suggests a hypothesis with respect to the mechanism underlying the association, which can be tested in experimental assays. However, there are a number of caveats to this approach that need to be correctly evaluated.

First, it must be noted that a large degree of non-functional overlap can be expected, because of the widespread distribution of the biochemical events. Thus, it is necessary to use unbiased approaches to evaluate which of the overlaps are functionally relevant and which occur by chance 13.

Second, regulatory elements that influence gene expression may operate in a spatial- and temporal-dependent manner 14. Therefore, annotation data should ideally be retrieved for a cell type and developmental stage that is most relevant to the trait under investigation. For example, genetic signals associated with type 2 diabetes may be annotated using data sets obtained in pancreatic islets 13,15. However, for many complex traits including common diseases, the identity of relevant cell types is not obvious (e.g. for height or longevity), or appropriate cell types and tissues are difficult – or even impossible – to obtain for experimental assays (e.g. cells from the cerebral cortex in Alzheimer's disease). Several common disorders have been associated with distinct developmental phases such as fetal stages in metabolic syndrome and adult stages in age-related diseases 16. Indeed, a recent study showed enrichment of GWAS signals associated with cardiovascular disease at regulatory regions in fetal tissue, and depletion of signals linked to breast cancer and Alzheimer's disease 11.

More and more genome annotation data sets are becoming available, including those created by the Encyclopedia of DNA Elements (ENCODE) Project Consortium 12, NIH Roadmap Epigenomics Mapping Consortium 17, and the recently launched BLUEPRINT Consortium 18. While the ENCODE data comprises mainly transformed cell lines (e.g. due to practical reasons such as their wide availability and capacity to produce large numbers of cells), the Roadmap Epigenomics and BLUEPRINT data almost exclusively consist of a broad selection of primary, ex vivo tissues corresponding to normal tissues and organ systems that are involved in human disease processes. These consortia have also contributed towards setting standards for experimental protocols, reagents, and bioinformatic tools. In particular, genome browsers are valuable for accessing and visualizing the consortia's multi-dimensional data sets 19,20.

The growing number of genome annotation data sets will enable the functional interpretation of GWAS signals in an increasingly context-specific manner. Alongside these, it will be necessary to refine computational methods to distil the vast amount of data into discrete segments of interpretable biological function. Segmentation approaches determine patterns and similarities between individual chromatin data sets to summarize them into a small set of “chromatin states” 21–23. Nonetheless, the functionality of the predicted chromatin states, such as “strong” and “weak enhancer”, as well as their predicted cell type specificity, need experimental validation.

## Choosing a suitable cellular system for the annotation of GWAS loci

**Figure 1:**
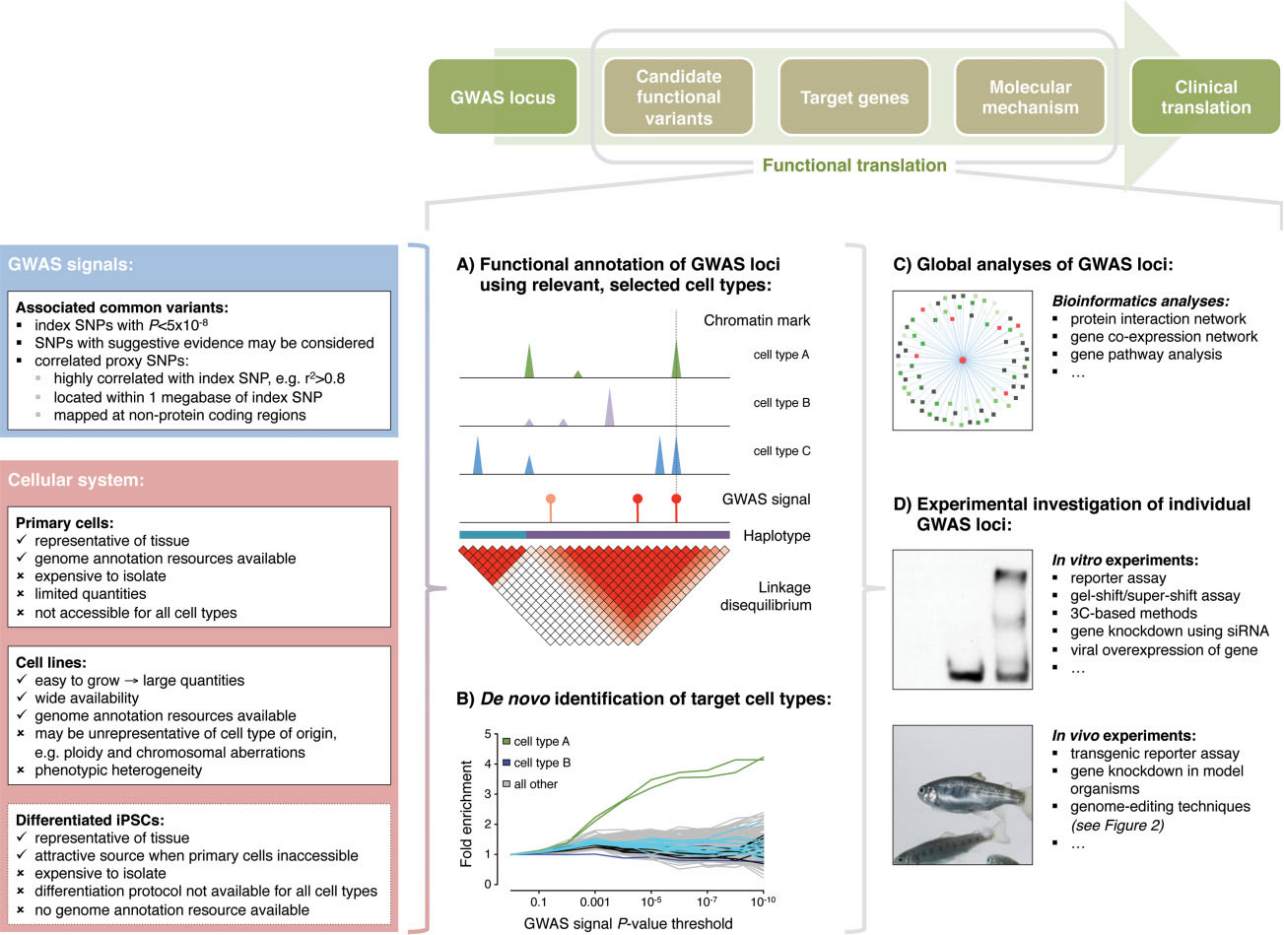
Genome annotation data in different cellular systems guide the functional interpretation of genetic variation. The pros and cons of annotation data sets obtained in different cellular systems are indicated on the left panel. Currently, publicly available resources mainly consist of data sets derived from primary cell cultures and transformed cell lines, while iPSCs may become more prominent in the future. Indeed, iPSC-derived cells may represent the key technological advance for assaying inaccessible cell types for which primary cultures cannot be obtained. Functional annotation of genetic signals at GWAS intervals can be restricted to selected cell types that are most relevant to the trait of interest (A), or unrestricted using all available cell types in an annotation resource (B). The latter approach may be valuable if target cell types of the trait are not yet established. To gain biological insights into the genetic architecture of complex traits, all GWAS signals may be collectively analyzed and associated with gene pathways and networks using bioinformatic tools (C). In parallel, individual GWAS intervals may be studied in depth using a range of in vitro and in vivo experimental assays (D). The use of emerging genome-editing techniques is illustrated in detail in Fig. 2. Abbreviations: LD, linkage disequilibrium; siRNA, small interfering RNA; TALEN, transcription activator-like effector nuclease; CRISPR, clustered regularly interspaced short palindromic repeats; 3C, chromosome conformation capture.

Genome-wide annotation data sets can be obtained in several cellular systems, mainly from primary cell cultures and transformed cell lines, while in the future, differentiated iPSCs are likely to become more prominent for this application 24. Each cellular system has its pros and cons when applied to the prioritization of candidate regulatory variants at GWAS loci (Fig. 1).

Primary cells are directly representative of the tissues and organs from which they were isolated. However, the isolation of homogeneous cell populations is challenging, involving preparative procedures such as fluorescent-activated sorting of cells. Therefore, many primary cell populations are available in limited quantities for experimental assays. In addition, chromatin maps generated in primary cells and tissues represent only a snapshot of the development stage during which they were isolated.

Cultured cell lines and transformed (immortalized) cell lines mostly retain the characteristics of the primary tissue from which they were derived. However, chromosomal rearrangements, changes to chromatin structure, DNA methylation, and gene expression profiles may arise through, for example, serial subculturing of the cell lines 25. These non-physiological transformations can lead to the appearance of artificial biochemical activities and misleading annotation of genetic variants. Indeed, we recently compared enrichment patterns of hematological trait-associated variants at sites of open chromatin in a selection of primary hematopoietic cells and their representative cell lines 26. We observed a much larger number of both open chromatin sites and overlaps of these sites with trait-associated variants in cell lines compared to primary cells. The enrichment patterns in cell lines showed decreased specificity with respect to cell type identity.

The prioritization of candidate regulatory variants at GWAS loci by means of publicly available annotation resources suffers from a trade-off between sensitivity and specificity 27. That is, GWAS signals can be annotated using functional genome maps derived from either selected cell types that are of most relevance to the phenotype of interest (Fig. 1A) or from across all available cell types (Fig. 1B). Limiting the annotation to selected cell types may only be considered if extensive prior knowledge about the target cell and tissue type exists. For example, this applies to some extent to the cellular components of the well-characterized hematopoietic system 26,28. Conversely, de novo identification of target cell types (i.e. out of all available cell types collected in an annotation resource) has emerged as key application of the functional annotation of GWAS signals and has already revealed novel target cell types for a number of common diseases. These include IL-17-producing T helper (TH17) cells in Crohn's disease and CD19^+^/CD20^+^ B cells in multiple sclerosis. Indeed, this approach may recognize pathogenic cell types without prior knowledge of disease-relevant molecular processes 11.

## “Scientists, choose your weapons”

Besides choosing a cellular system that is biologically relevant for the screening of candidate functional variants at GWAS loci, as outlined above, attention has to be paid to choosing a suitable genome annotation mark. In this respect, we advocate the use of a *general* hallmark of regulatory potential, such as open chromatin provided by deoxyribonuclease I (DNase I)-seq, formaldehyde-assisted isolation of regulatory elements (FAIRE)-seq, or the recently introduced assay for transposase-accessible chromatin (ATAC)-seq 29, as opposed to an *individual* mark, such as transcription factor binding provided by chromatin immunoprecipitation (ChIP)-seq.

Sites of open chromatin are associated with most classes of active gene regulatory elements, and as such, are specific to cell type, developmental stage, and other influencing factors 30,31. In contrast, in transcription factor ChIP-seq experiments, antibodies against a distinct DNA-binding protein are applied. The application of open chromatin as a screening tool for candidate functional variants is both informative and cost-effective, because one would need to obtain a substantial number of transcription factor ChIP-seq experiments to obtain a comparable information value. Besides, ChIP-seq data thus far generated by the ENCODE Consortium are scarce with respect to the number of cell types in which most transcription factors have been assayed. In addition, antibodies against particular transcription factors may not be available or validated for ChIP. The drawback of open chromatin assays is their lack of specificity, as the identified sites typically correlate with the binding sites of many different transcription factors 32. Therefore, additional annotation data sets are required to verify the presence and type of a regulatory element (e.g. enhancer vs. promoter) and transcription factor-binding site. Here, in silico predictions may guide the identification of the specific transcription factor involved.

Alternatively, ChIP-seq of components of the gene expression machinery may also be useful for the annotation of candidate regulatory variants. For example, ChIP-seq of the transcriptional co-activator protein p300 has been shown to associate with active, cell type-specific enhancer sequences 33. ChIP-seq of histone modifications that mark active promoters and enhancers are informative, but their typical broader peaks may impede precise screening and identification of candidate functional variants.

## Picking strategies for the prioritization of candidate functional variants

To gain biological understanding of the genetic variants associated with complex traits including disease susceptibility and outcome, at least two strategies have been pursued. First, the combined analysis and interpretation of all genetic signals identified in a GWAS (Fig. 1C) and second, the in-depth analysis of selected, individual GWAS loci (Fig. 1D). The two strategies are described in detail in the following sections.

### Global analyses of GWAS loci

Much biological insight from GWAS can be gained when multiple association signals are collectively co-analyzed. Rather than aiming to identify distinct molecular mechanisms, these bioinformatic approaches focus on connecting a selection of target genes and their products with knowledge databases, e.g. concerning tissue-specific gene expression signature, intracellular localization, citation in the literature (PubMed abstracts) or gene ontology terms 34–36. Potential target genes in proximity to all index SNPs (*p* < 5 × 10^−8^) or a subset of SNPs reaching a certain significance threshold (e.g. *p* < 0.05) may be tested for association with shared gene pathways and networks. Of course, identified associations are not necessarily causal and require further validation. Findings may also be biased towards well-studied and frequently reported gene pathways. Nonetheless, these bioinformatic tools may provide a powerful means of generating novel hypotheses regarding molecular processes involved in disease etiology. For example, an integrated pathway analysis approach recently highlighted the striking role for host responses to mycobacteria in inflammatory bowel disease 37.

Annotating GWAS signals with information on gene expression can yield a better understanding of the regulatory networks underlying the association. In studies on expression quantitative trait loci (eQTLs), the alleles of the index or proxy SNP are correlated with variation in transcript levels that are quantitatively measured in unrelated individuals using gene expression arrays or – more sensitively and accurately – RNA-seq. SNPs that show a strong correlation with expression levels for a specific gene are likely to mark an eQTL for that gene. Both local- and distal-acting eQTLs can be identified, but the identification of distal-acting eQTLs has been largely unfruitful due to the inherent limited statistical power of the approach 38. Systematic studies have demonstrated the practicality and efficiency of eQTLs as screening tool for candidate regulatory variants 39,40. As a relatively large number of individuals need to be sampled to gain statistical confidence in the observed association, easily accessible cell types (e.g. lymphoblastoid cells or monocytes) or cell types that have been extensively characterized, are usually chosen over the most relevant ones. However, it is important to note that a substantial proportion of eQTLs identified are cell type-restricted 41. Furthermore, SNPs associated with transcript levels may not be causal, as eQTL studies suffer from LD structure. Although most eQTL studies have focused on protein-coding RNAs, non-coding RNAs (i.e. large intergenic non-coding RNAs; lincRNAs) are equally relevant candidates 42.

Instead of measuring gene expression levels across individuals, allele-specific expression (ASE) analysis measures transcript abundance within an individual 43. In this powerful approach, transcript levels are assessed using RNA samples derived from individuals that are heterozygous at a particular eQTL SNP of interest. Transcripts that deviate from the expected 1:1 ratio at heterozygous alleles (i.e. show “allelic imbalance”) are likely candidate transcripts. For example, ASE analysis has been applied to the asthma and autoimmune disease risk locus on chromosome 17q12-q21 pinpointing potential causal sequence variants 44.

### Experimental investigation of individual GWAS loci

Ascertaining an exhaustive account of common as well as low-frequency variants is an important, initial step in the in-depth molecular analysis of selected, individual GWAS intervals. However, only a fraction of all genetic variants are examined in GWAS. Genotype imputation exploits known LD patterns and haplotype frequencies from reference data sets to estimate genotypes for additional SNPs not directly assayed in the initial genome-wide scan 45. In addition to genotype imputation, established association intervals may be fine-mapped using dense, custom genotyping arrays. Such arrays are based on the deep sequencing catalogue of the 1,000 Genomes Project 10 and contain essentially all common and low-frequency variants at selected GWAS loci of a group of related clinical conditions such as autoimmune and inflammatory diseases 46,47. Selected GWAS intervals may be resequenced to enable sequencing-based genotyping. However, the costs for such an experiment may be considerable (e.g. due to the relatively deep coverage and large number of subjects required). We therefore argue for the application of sequencing data from the 1,000 Genomes and UK10K Projects (http://www.uk10k.org/), which should give sufficient account of low-frequency and rare sequence variation.

After the comprehensive assessment of genetic variation at GWAS intervals, the variants are then overlapped with genome annotation marks in order to identify candidate functional variants. Importantly, mere annotation of genetic variants using epigenomic data sets does not prove molecular function and causality (that is, impact on organismal phenotype). Candidate regulatory variants that overlap with one or more annotation marks require substantial experimental validation. This should involve an integrated approach of multiple experimental methods to gain confidence in the observed effect. Most frequently applied in vitro cellular assays include luciferase reporter assays 15,48–51, gel-shift and super-shift assays 49,50,52, as well as allele-specific chromatin assays 44,53, which should be performed in relevant cell types to avoid misleading biological interpretations (see above). Candidate regulatory sites may also be tested using in vivo assays. For example, the activity of tissue-specific enhancer sequences can be assessed in transgenic mouse assays 54.

Regulatory variants potentially lie great distances from the gene(s) they control, functioning through long-range regulatory interactions 11,55,56. Chromosome conformation capture (3C) and descended methods (e.g. circular 3C, 4C; enhanced 4C, e4C; and Hi-C), as well as chromatin interaction paired-end tagging (ChIA-PET) techniques, examine long-range physical interactions between distal gene regulatory elements and promoter regions of target genes and have already been successfully applied to non-coding GWAS signals 11,55,56. These experimental tools, while technically challenging to perform, provide an unprecedented view of the interplay between regulatory variants and genes at GWAS intervals.

Once established, target genes may be further characterized with respect to the trait of interest. Here, traditional assays to characterize gene function can be used including gene knockdown using small interfering RNA (siRNA) or gene overexpression using adeno-associated viral vectors 50. For a number of complex traits, e.g. hematological traits such as platelet counts and volume, gene knockdown in zebrafish (*Danio rerio*) embryos using morpholinos (antisense oligonucleotides) has proved particularly insightful. In systematically switching-off candidate genes at GWAS intervals, several novel genes implicated in platelet formation were identified and successfully validated 57.

Investigating the molecular mechanism of individual GWAS loci is arduous, and arguably, scalable in vitro approaches are needed to experimentally validate candidate functional variants in a high-throughput manner. Indeed, progress has been made in massively parallel reporter assays, which use large-scale DNA synthesis and next-generation sequencing to simultaneously measure the reporter activity of many thousands of enhancer variants 58,59.

## Revolutionizing the functional translation of GWAS signals by genome engineering

Despite remarkable technological advances for the discovery of genetic variation, the experimental tools to study the molecular mechanisms of candidate functional variants have seen only little progress thus far. To this end, we envisage the application of site-specific genome-editing techniques to be game-changing. Transcription activator-like effector nucleases (TALENs) 60–63 and clustered regularly interspaced short palindromic repeats (CRISPR)/CRISPR-associated (Cas) systems 64,65 are novel classes of genome-editing techniques. These methods enable the modification of any genomic sequence of interest in mammalian cells and model organisms.

TALENs comprise a FokI nuclease domain, which cleaves DNA in a non-sequence-specific manner, fused to a modular DNA-binding domain. The DNA-binding domain is composed of highly conserved amino acid repeats, transcription activator-like effectors (TALEs), which can be engineered to recognize specific DNA sequences. The engineered nucleases bind as a dimer to a target site, where they induce a DNA double-strand break. In turn, DNA damage response pathways are triggered, such as non-homologous end-joining (NHEJ) or homology-directed repair (HDR), which enable the precise introduction, exclusion or alteration of gene alleles at the target site. Bauer et al. applied TALENs to modulate the activity of an enhancer sequence critical for erythroid expression of *BCL11A*, a gene implicated in hemoglobin disorders 66,67. The lineage-specific enhancer contains common sequence variants identified through GWAS, which impact erythroid transcription factor binding. The authors suggest this GWAS-identified enhancer as potential therapeutic target in hemoglobinopathies.

**Figure 2:**
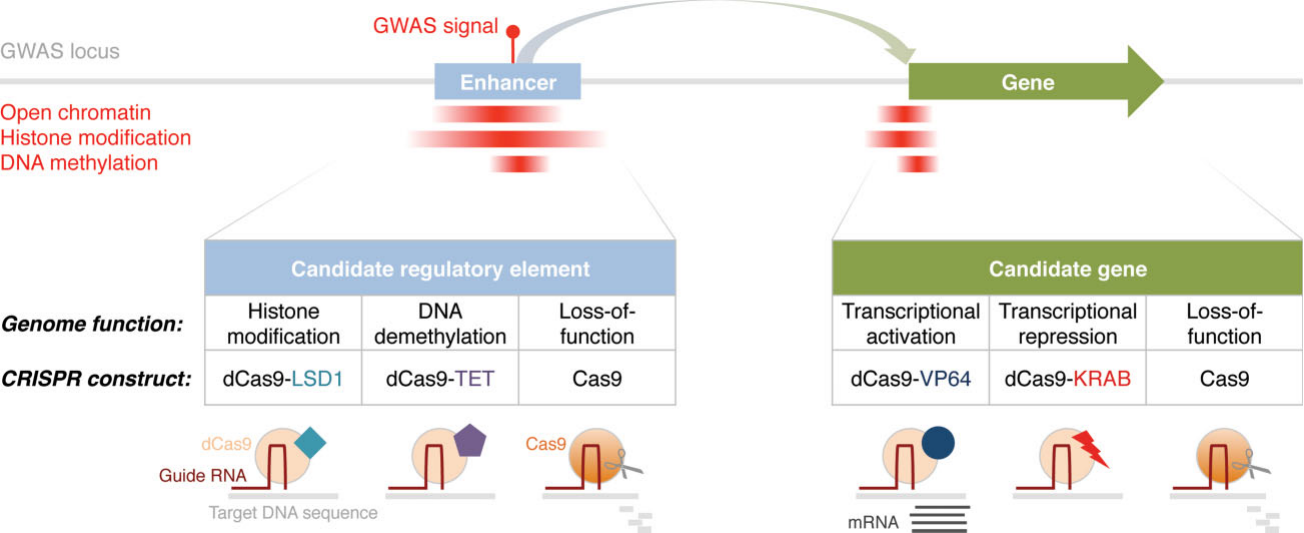
Investigating molecular consequences of candidate functional variants using CRISPR/Cas9. The advent of novel genome-editing techniques, such as CRISPR/Cas9, enables exciting new opportunities for validating GWAS candidate regulatory sites and genes. CRISPR/Cas9 in conjunction with customizable guide RNA can be used to precisely target genomic sites of interest to induce loss-of-function alterations. In addition, CRISPR-associated catalytically inactive Cas9 protein (dCas9) can be fused to different effector domains, including VP64 (activation), KRAB (repression), LSD1 (histone demethylation, specifically H3K4me2 and H3K27ac), and TET family proteins (DNA demethylation). Upon introduction of the CRISPR/(d)Cas9-complex into a cellular system, the molecular consequences of the genome editing can be further investigated.

CRISPR/Cas systems have recently emerged as an alternative to TALENs, vastly improving its cleavage efficiency and ease of implementation at reduced cost 65,68. Type II CRISPR/Cas systems use Cas9 nucleases that are guided to a genomic sequence of interest via synthetic RNA molecules. Thus, CRISPR's application of guide RNAs supersedes the need for engineering custom proteins. In addition to the disruption of genomic sequence through nucleases, CRISPR/Cas9 may be provided with effector domains that exert distinct regulatory functions. For example, the CRISPR-associated catalytically inactive Cas9 protein, termed dCas9, can be fused to activator domains 69,70, repressor domains 69 or potentially domains that alter different epigenetic states 71. Such modified CRISPR/dCas9-fusion proteins, together with guide RNA, can then be introduced to control the activity of candidate regulatory elements that harbor GWAS signals or candidate genes. In Fig. 2, we have summarized different types of CRISPR/Cas9 constructs and their potential application to examine regulatory elements at GWAS intervals.

For genome-editing techniques, the issue of specificity and delivery is paramount. The desired genome modification should ideally occur with high frequency in the cell population and with no detectable off-target effects. Although rapid progress has been made in this respect 72, further improvements in molecular design and experimental procedures are needed to please sceptical reviewers. Here, we suggest light-inducible transcriptional effectors (LITEs) to offer exciting possibilities for studying the function and regulation of mammalian genomes in the near future. LITE modules consist of the light-sensitive photoreceptor cryptochrome 2 (CRY2) that is fused to a customizable DNA-binding domain based on the TALE or CRISPR/Cas9 systems. The construct allows spatially and temporally precise, graded, reversible and non-invasive modulation of gene transcription and epigenetic states 73,74.

CRISPR/Cas9 constructs can be introduced into human somatic cells and iPSCs (via transfection) as well as various model organisms (e.g. via injection of mouse zygotes 75). For application in human somatic cells, we suggest the use of cells of defined genotype. Resources of healthy individuals, who can be recalled on the basis of their genotype for donating cellular components (e.g. immune effector cells) and subsequent functional studies, have proven valuable 51,76. However, we advocate the use of CRISPR/Cas9 in conjunction with iPSC technology. iPSCs are generated by reprogramming mature adult cells, such as fibroblasts, into cells that hold properties of embryonic stem cells. From this state of immaturity, iPSCs have the potential to differentiate in vitro into a wide range of specialized cell types. For example, detailed protocols have been published describing the differentiation of iPSCs into human cerebral cortex neurons 77,78 – cells that cannot be obtained from primary sources. By differentiating iPSCs into distinct lineages, the effect of CRISPR/Cas9 can be assessed under different genetic programmes in the same cellular system. For example, an annotated regulatory element containing a GWAS signal associated with Alzheimer's disease may be disrupted using CRISPR/Cas9 in iPSCs. Following differentiation of the iPSCs into cerebral cortex neurons, the functional consequences of that genome alteration can be compared with the control iPSC population, e.g. through transcriptome analysis. Furthermore, multiple implicated GWAS signals, regulatory elements and genes can potentially be disrupted in combination to examine their interaction or synergistic effects. Lastly, this experimental design may be expanded to iPSCs derived from patients, enabling the possibility to “cure” disruptive effects of GWAS signals at the cellular level. The latter may be boosted by, for example, the newly established UK Human Induced Pluripotent Stem Cells Initiative (HipSci; http://www.hipsci.org/). This open-access resource will create iPSC lines derived from over 1,000 healthy individuals and individuals with genetic diseases by 2016. Moreover, the proposed approach would make currently available tools for the correction of disease-causing mutations more efficient, such as zinc finger nucleases (ZFNs) combined with piggyBac transposons in iPSCs 79.

## Striving towards the functional interpretation of rare non-coding sequence variation

Low-frequency (MAF, 1–5%) and rare (MAF < 1%) genetic variants (not captured by GWAS) may explain a substantial fraction of the genetic component of complex traits including common diseases 80,81. Costs of next-generation sequencing applications have plummeted over the last years and with innovative sequencing methods on the horizon, notably nanopore DNA sequencing, genotyping-based methods will likely be replaced by sequencing-based methods for detection of trait-associated variants. Arguably, this will entail not only challenges for the design of association analyses of low-frequency and rare alleles linked to complex traits and diseases (e.g. extreme trait resequencing or family-based studies 80), but also for the functional interpretation of the implicated alleles. Compounded by both allelic and locus heterogeneity 82, the sheer number of low-frequency, rare and private variants will make identification of the causal variants challenging. To increase statistical power, studies thus far have therefore centred exclusively on variants of apparent functional consequence, i.e. missense, nonsense, frame-shift or splicing variants. However, it can be expected that some of the causal sites will reflect gene regulatory variants 83. Akin to the functional classification of coding variants, non-coding variants may be recognized as, for example, disrupting transcription factor binding sites. However, such sites are usually around 200 bp in size when identified using ChIP-seq and sometimes only a fraction of these sites harbor the known binding motif of the transcription factor. In contrast, nucleotide-resolution techniques, such as DNase I and ATAC-seq footprinting, allow for identification of the exact location of the transcription factor binding site 29,84. Therefore, we argue that these assays in particular are ideally suited to systematically prioritize low-frequency and rare alleles, due to the reduction of sequence space with likely biological importance. However, we note that in contrast to coding regions where rare variants that are likely to be deleterious can be combined for statistical testing, it is currently unclear how to categorize and test a large number of rare variants across regulatory regions with uncertain functional consequences.

## Conclusions and outlook

We believe there is still substantial scope for performing GWAS in the coming years. Well-powered meta-analyses of GWAS detect novel small-effect association regions, and fine-mapping approaches as well as studies in ethnic subgroups refine existing ones. In parallel, we call for a boost in the number of investigations into the molecular mechanisms of confirmed associations. There is a pressing need to translate genetic signals of complex traits and diseases into molecular mechanisms, through both global meta-analysis of multiple GWAS intervals and in-depth mechanistic studies of transcription, chromatin structure and DNA methylation at individual GWAS intervals. This functional translation is crucial for the identification of novel “druggable” or reversible components and pathogenic pathways. This in turn has the potential to empower clinical care through, for example, improved risk prediction, biomarker identification, disease subclassification, drug development and dosing 85.

Despite the identification of over 2,000 robust associations with more than 300 complex traits and diseases 85, only a negligible fraction of discovered GWAS intervals have been followed up in experimental studies. In most cases, the causal variant(s) as well as gene(s) are unknown. With the advent of clinical (http://www.genomicsengland.co.uk/) and personal (http://www.personalgenomes.org/) whole-genome sequencing, an overwhelming number of sequence variants will be identified at non-coding regions with potential regulatory effects. Thus, efficient strategies for functional translation are urgently needed. To this end, we expect that CRISPR/Cas9 will play a pivotal role in unravelling molecular mechanisms for a significant number of trait-associated genetic variants. Although not discussed in detail in this article, we suggest that proteomic tools could also be integrated in the functional translation of GWAS findings. For example, the interaction dynamics between a trait-associated variant and a transcription factor complex may be characterized by mass spectrometry 86. It is important to note that the subtle phenotypic effects of trait-associated variants discovered through GWAS should not be inferred as indicative of subtle effects of the regulatory element at which they reside. In contrast, they may potentially have a strong phenotypic effect as part of a gene regulatory complex 66.

Non-genetic factors, e.g. epigenetic variation, have been suggested to have a substantial impact on complex trait etiology. Similar to GWAS, such epigenetic variation (specifically, DNA methylation) can be assayed across many individuals and tested for association with a complex trait of interest in epigenome-wide association studies (EWAS) 87. EWAS may be used to explore genetic risk alleles that mediate their effects through epigenetic mechanisms 88,89. Thus, integration of GWAS and EWAS presents a promising strategy to unravel the underlying biological mechanisms of complex traits and diseases 90–92. Here, the newly formed Genetics of DNA Methylation Consortium (GoDMC; http://www.godmc.org.uk/) will bring together scientists studying the genetic basis of DNA methylation and provide a centralized hub for coordinating data analyses.

## Abbreviations

ChIP: chromatin immunoprecipitation
CRISPR: clustered regularly interspaced short palindromic repeats
ENCODE: Encyclopedia of DNA Elements
eQTL: expression quantitative trait locus
GWAS: genome-wide association study
iPSC: induced pluripotent stem cell
LD: linkage disequilibrium
MAF: minor allele frequency
SNP: single-nucleotide polymorphism
TALEN: transcription activator-like effector nuclease
